# Malignant Priapism as the Sole Initial Manifestation of Metastatic Carcinoma with Suspected Pulmonary Origin: A Case Report and Review of the Literature

**DOI:** 10.3390/reports9030317

**Published:** 2026-09-20

**Authors:** Sara Riolo, Dario Brunello, Agostino Fraia, Salvatore Papi, Roberto Knez, Giovanni Costa, Bernardino De Concilio, Tommaso Silvestri, Ivan Di Giulio, Guglielmo Zeccolini, Antonio Celia

**Affiliations:** 1 Department of Urology, Sapienza University of Rome, 00185 Rome, Italy; 2 Urology Unit, Department of Surgery, Dentistry, Pediatrics and Gynecology, Azienda Ospedaliera Universitaria Integrata, University of Verona, 37126 Verona, Italy; 3 Department of Neurosciences, Reproductive Sciences and Odontostomatology, University of Naples “Federico II”, 80131 Naples, Italy; 4 Department of Urology, San Bassiano Hospital, 36061 Bassano del Grappa, Italy

**Keywords:** malignant priapism, lung cancer, pulmonary adenosquamous carcinoma, penectomy

## Abstract

**Background and Clinical Significance:** Malignant priapism is a rare manifestation of advanced malignancy caused by malignant infiltration of the corpora cavernosa. We report a case of persistent and refractory priapism associated with penile carcinoma and extensive metastatic disease, with a suspected pulmonary primary. **Case Presentation:** An 81-year-old man with a history of prostate cancer treated by radical prostatectomy and persistently undetectable PSA levels developed persistent low-flow priapism. Despite aspiration and irrigation, followed by distal and proximal shunting procedures, the condition recurred with progressive corporal induration and severe pain. Cavernous biopsy was initially interpreted as moderately differentiated squamous carcinoma, while total penectomy performed for refractory pain showed poorly differentiated carcinoma with morphological adenosquamous features. CT and PET/CT demonstrated a pulmonary mass and widespread metastatic disease, although bronchoscopy was non-diagnostic and no histological confirmation of the pulmonary lesion was obtained. A focused review of the literature identified 32 published cases of malignant priapism; bladder cancer was the most frequently reported primary malignancy (37.5%), followed by prostate cancer (18.8%) and renal cell carcinoma (9.4%), while penectomy was performed in 34.4% of cases. **Conclusions:** Persistent or recurrent priapism associated with corporal induration, progressive or atypical pain, failure of conventional treatment, or discordant imaging findings should raise suspicion of malignant infiltration and prompt consideration of repeat imaging and tissue sampling. In the present case, carcinoma involving the penile structures was histologically confirmed, whereas pulmonary origin remained a clinicoradiological suspicion; penectomy provided immediate pain relief in the setting of extensive refractory local disease.

## 1. Introduction and Clinical Significance

Malignant involvement of the penis is an exceptional clinical finding despite the organ’s rich vascular and lymphatic supply. Penile metastases account for a very small proportion of secondary malignancies, with fewer than 500 cases reported in the literature to date [1]. Most metastatic lesions originate from pelvic organs, particularly the bladder and the prostate, followed by the gastrointestinal tract, whereas extrapelvic primaries such as kidney or lung cancer are distinctly rare [2].

Malignant priapism, defined as a persistent erection caused by neoplastic infiltration of the corpora cavernosa, represents one of the least common clinical presentations of penile malignancy. First described by Peacock in 1938 [3], malignant priapism is typically associated with advanced-stage disease and carries a dismal prognosis [4]. Importantly, patients with penile metastases from non-urological primary malignancies appear to have a poorer prognosis than those with metastases from urological tumours. Furthermore, the presence of malignant priapism has been associated with an even worse prognosis compared with penile metastases presenting without priapism, highlighting the particularly aggressive nature of this clinical presentation [4].

The main mechanisms of penile metastasis include retrograde venous spread, facilitated by the extensive communication between the pelvic venous plexuses and the penile dorsal venous system; retrograde lymphatic dissemination; and direct extension from adjacent organs in the setting of locally advanced disease. Haematogenous spread through the arterial circulation represents a less common route. Malignant priapism may occur when metastatic infiltration of the corpora cavernosa obstructs the cavernous venous outflow while preserving arterial inflow, resulting in venous congestion and subsequent ischaemic priapism [5].

Primary carcinoma of the penis is itself uncommon, with squamous cell carcinoma accounting for the vast majority of cases. Adenosquamous carcinoma is a particularly rare histological variant, characterized by the presence of both glandular and squamous components and by an aggressive biological behavior [6]. Even rarer is secondary penile involvement by adenosquamous carcinoma arising from distant organs, including the lung.

Adenosquamous carcinoma of the lung represents a rare subtype of non-small cell lung cancer, accounting for approximately 0.4–4% of cases, and is associated with rapid progression, early metastasis, and poor survival compared to pure adenocarcinoma or squamous cell carcinoma [7].

We present a rare case of malignant priapism as the sole initial manifestation of aggressive metastatic carcinoma, with a suspected pulmonary primary, in a patient who was otherwise asymptomatic from a respiratory and systemic standpoint. The diagnostic evaluation, treatment, and overall management were challenging and required a multidisciplinary approach. In addition, we performed a focused review of the literature to contextualize our case within the spectrum of reported cases of malignant priapism, with particular attention to the underlying primary malignancies, clinical presentation, and therapeutic management. Through this case and review, we aim to highlight the potential malignant aetiology of recurrent priapism and raise awareness among clinicians of the importance of considering an underlying malignancy, particularly when conventional causes have been excluded.

## 2. Case Presentation

An 81-year-old man was referred to our urology department in August 2025 for persistent penile erection that had started in July 2025. A chronological summary of the diagnostic and therapeutic course is provided below (Table 1). His family history was notable for cardiovascular disease (father and sister died due to myocardial infarction) and a brother undergoing radiotherapy for lung cancer (unknown histology).

Past medical history included prostate cancer treated with robot-assisted radical prostatectomy in 2017, followed by regular oncological follow-up with serial PSA measurements consistently undetectable. Additional comorbidities included coronary artery disease treated with angioplasty 25 years earlier, type 2 diabetes mellitus and cholecystectomy complicated by postoperative hematoma requiring interventional radiology. He was a former smoker with an 80 pack–year history, having quit smoking in 1982. Functional status was preserved, and at admission, he denied weight loss, cough, dyspnea, hematuria, or constitutional symptoms.

The patient reported the initial appearance of a palpable penile shaft induration beneath intact skin, followed days later by the onset of priapism. The pain progressively became severe and stabbing, radiating to the right hemiscrotum. No urinary symptoms or systemic complaints were reported.

On first physical examination, the penis was rigid, tender, and diffusely indurated. No visible cutaneous lesions, ulcerations, or nodules of the glans, shaft, or prepuce were present. No inguinal lymphadenopathy was detected. Corporal blood aspiration and irrigation with 0.9% saline solution was performed, with initial resolution of the priapism. Arterial blood gas analysis was performed and showed findings consistent with ischemic priapism, with mild acidosis and hypercapnia. Bedside penile ultrasonography demonstrated a significant reduction in penile blood flow. Comprehensive laboratory testing for underlying hematologic disorders was carried out and yielded negative results.

A first penile MRI, performed early during the initial diagnostic work-up due to suspicion of local recurrence of prostate cancer, showed diffuse enlargement of the corpora cavernosa, particularly on the left, with diffuse signal abnormalities but no clearly defined focal lesion. The tunica albuginea and spongiosum appeared preserved, with no relevant lymphadenopathy. These findings were interpreted in the clinical context as compatible with ischemic priapism. Cystoscopic evaluation did not reveal evidence of a primary bladder tumor. Given the previous history of prostate cancer, the persistently undetectable PSA levels following radical prostatectomy also made recurrent prostate cancer less likely.

Despite initial conservative management, the patient experienced recurrent low-flow priapism and was again admitted to our ward. A distal bilateral T-shunt performed failed to achieve detumescence. Following another recurrence, a proximal corporospongiosal shunt was performed. During surgery, a cavernous tissue biopsy was obtained and initially interpreted as moderately differentiated squamous carcinoma.

A second MRI was performed and, compared with the previous examination, it demonstrated further enlargement of the corpora cavernosa, particularly on the left, with marked signal abnormalities and reduced central vascularization with predominantly peripheral enhancement. The urethral bulb was also enlarged, with peripheral enhancement. Signal abnormalities extended toward the ischiopubic branches, raising concern for involvement of the adjacent structures.

Due to suspicion of diffuse neoplasia, a total-body CT scan was requested. Thoracic CT revealed a 45 × 31 × 46 mm mass in the left lower lung lobe with diffuse interstitial thickening suggestive of lymphangitic carcinomatosis, multiple enlarged mediastinal lymph nodes, and small bilateral adrenal nodules. Bronchoalveolar lavage and repeated bronchoscopy with needle aspiration were non-diagnostic. Although these findings strongly suggested a pulmonary primary, no histological confirmation of the lung lesion was obtained.

**Figure 1:**
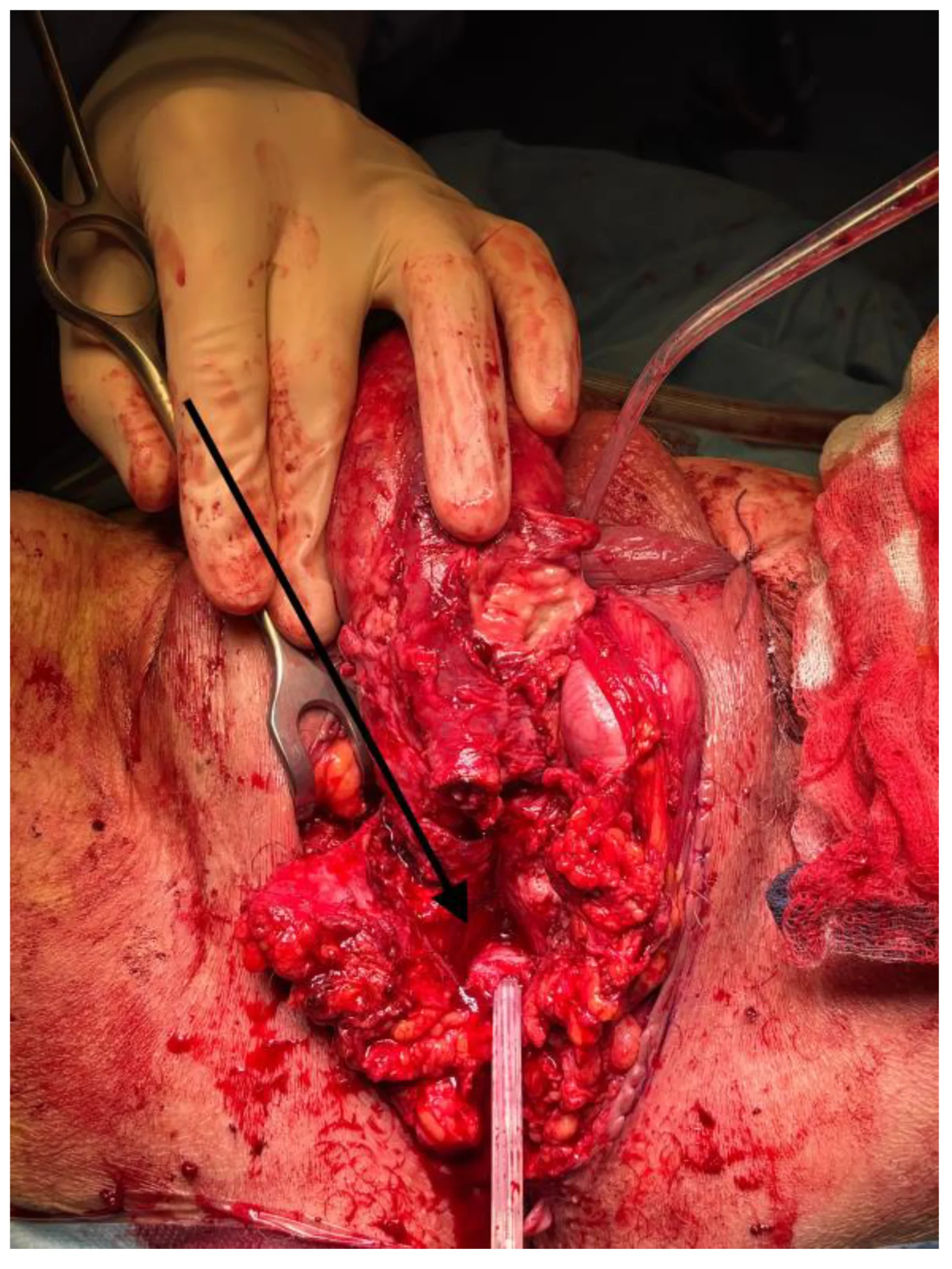
Intraoperative specimen showing section of the urethra prepared for perineal urethrostomy.

**Figure 2:**
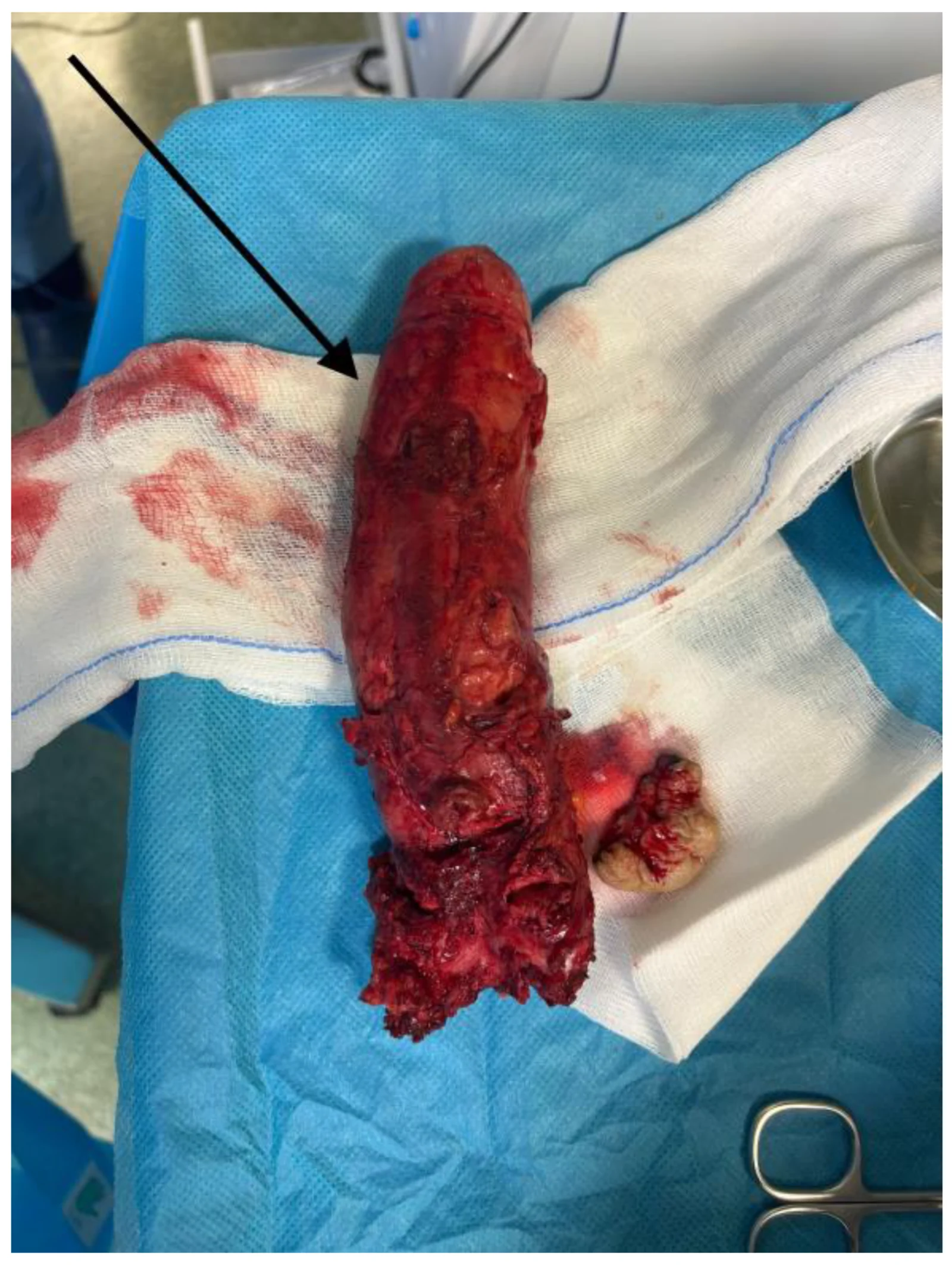
Gross surgical specimen demonstrating the resected penile tissue with marked enlargement and induration of the corpora cavernosa.

**Figure 3:**
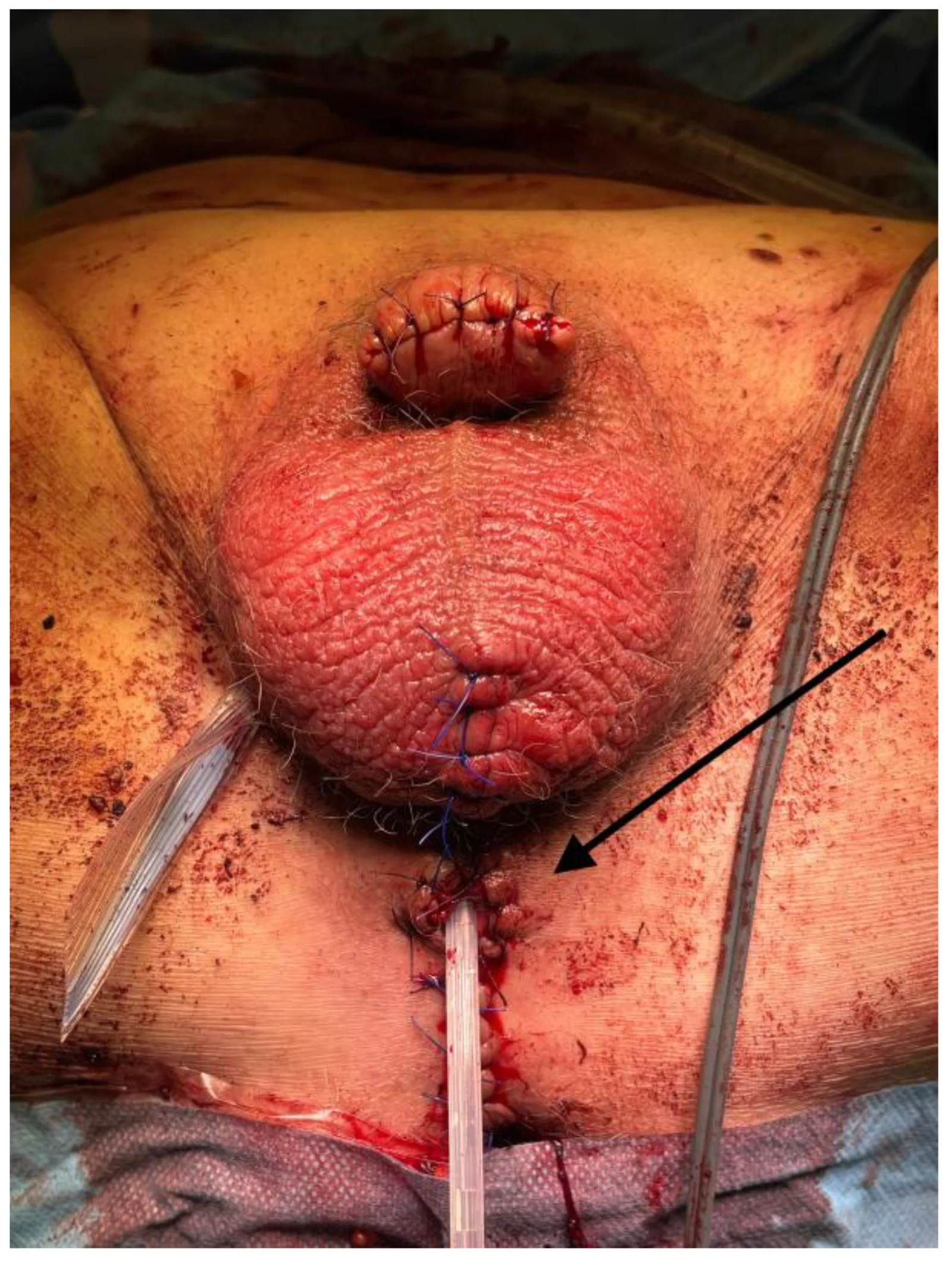
Final surgical reconstruction following total penectomy with partial skin preservation and perineal urethrostomy.

Given refractory pain, progressive necrosis, and failure of shunt procedures, total penectomy was performed on 13 November 2025. Intraoperatively, the corpora cavernosa were markedly enlarged, indurated, and necrotic. Penectomy with partial skin preservation, pedicled fat flap reconstruction, and perineal urethrostomy was carried out (Figure 1, Figure 2 and Figure 3).

Histopathology revealed a poorly differentiated carcinoma with adenosquamous features (G3), measuring 14 × 3.5 cm, extensively necrotic, with vascular and perineural invasion and positive surgical margins. The tumor showed morphological evidence of both glandular and squamous differentiation, although precise quantification of each component was not feasible due to extensive necrosis. Immunohistochemical analysis demonstrated positivity for CEA and CK7, while p63, p40, and p16 were negative. This immunoprofile was not entirely specific and did not allow definitive identification of the primary site. Additional markers that could support pulmonary origin, such as TTF-1 and Napsin A, were not performed, representing a limitation of the diagnostic work-up. Furthermore, the absence of p63 and p40 expression makes definitive confirmation of a squamous component challenging. Therefore, although the diagnosis of adenosquamous carcinoma was considered based on morphological features, alternative interpretations, including poorly differentiated adenocarcinoma, cannot be completely excluded. Pathological assessment demonstrated tumour invasion of both corpora cavernosa and the urethra, indicating extensive local spread within the penile structures.

Postoperatively, the patient experienced immediate and complete resolution of genital pain. The course was complicated by perineal wound dehiscence requiring surgical revision and by a non-infected sacral pressure ulcer.

**Figure 4:**
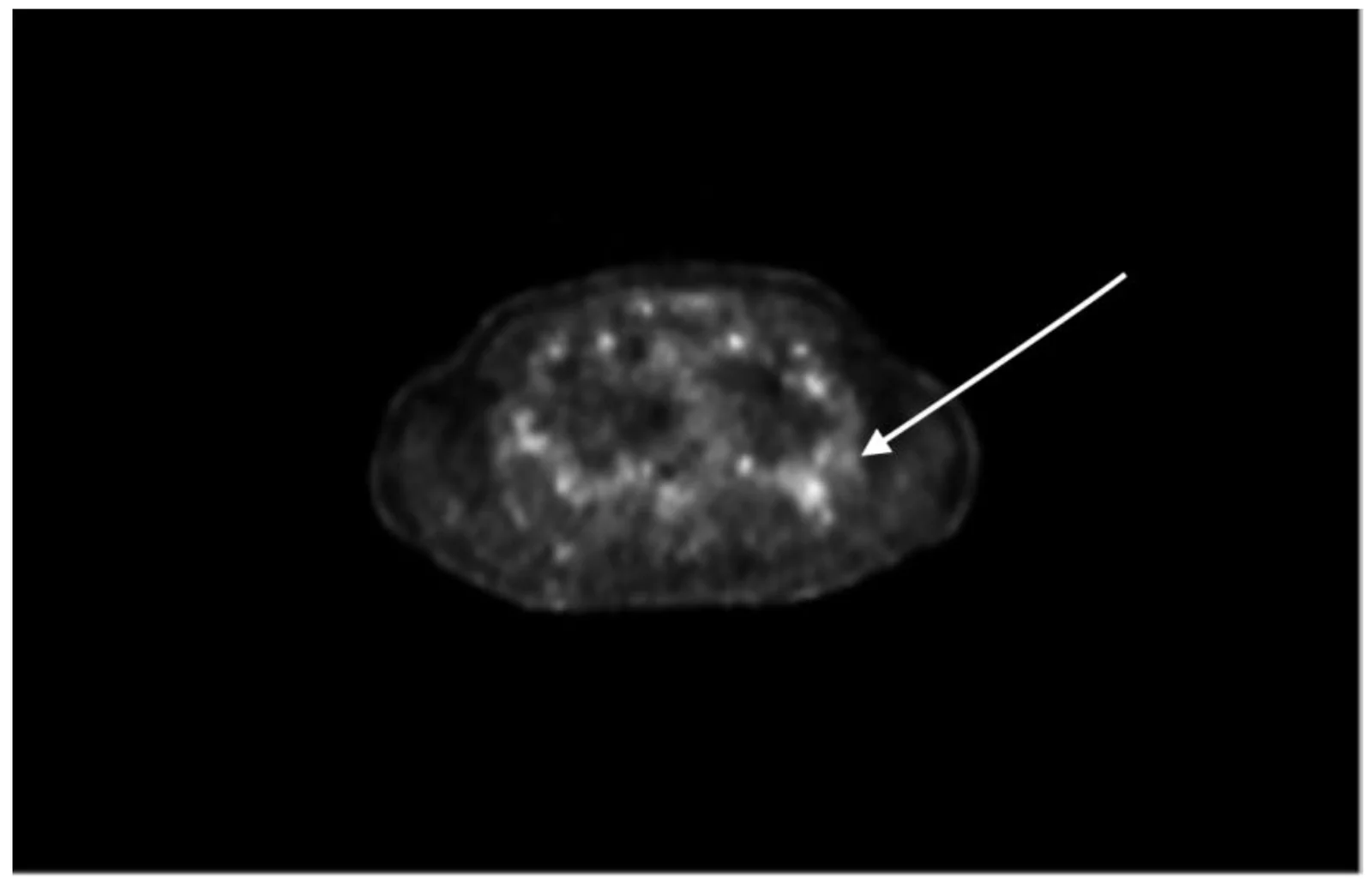
PET/CT imaging demonstrating a hypermetabolic mass in the left lower lung lobe (arrow), consistent with the suspected primary tumor.

**Figure 5:**
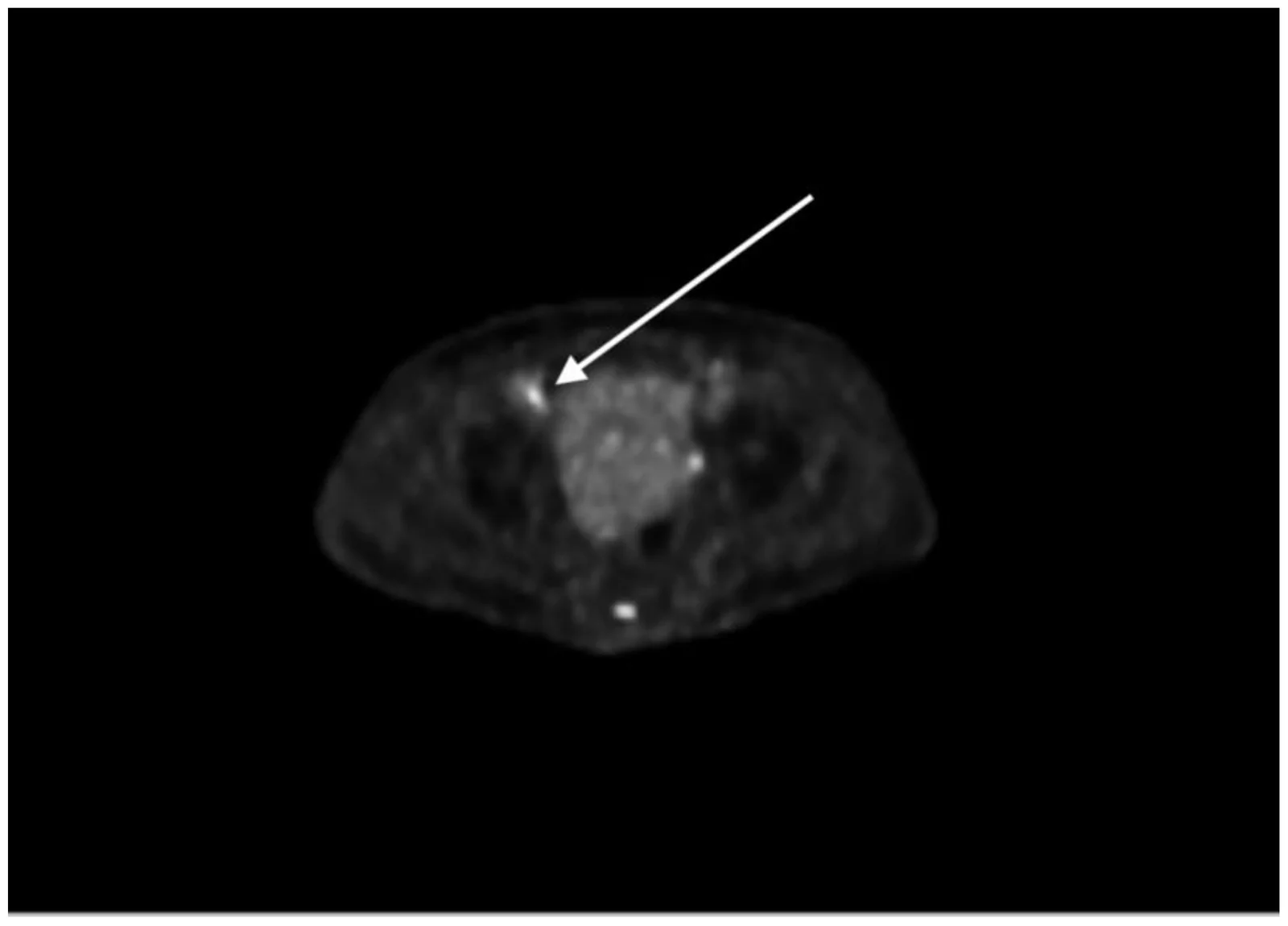
PET/CT imaging showing extensive pelvic and iliac lymph node involvement (arrow), consistent with metastatic disease.

Whole-body PET-CT demonstrated intense hypermetabolic activity in the left lower lung mass (SUVmax 10.4), extensive mediastinal, pelvic, and iliac lymphadenopathy, widespread bone metastases, soft-tissue deposits, and residual pelvic uptake consistent with cavernous involvement. PET/CT also has a recognized role in the literature in facilitating the diagnosis of secondary penile lesions [8]. Figure 4 and Figure 5 illustrate, respectively, the pulmonary involvement and the pelvic–lymph node involvement.

Given the aggressive disease, absence of diagnostic lung histology, advanced age, and rapid respiratory deterioration with hypoxemia, the patient was deemed unsuitable for systemic oncological treatment and was placed on best supportive care according to multidisciplinary oncological recommendation. Within a few days following PET-CT, the patient died due to worsening of the pulmonary condition; despite a request for autopsy by the medical staff, authorization was not granted by the family.

This case report was prepared in accordance with the CARE guidelines.

## 3. Malignant Priapism: Review of the Literature

Malignant priapism represents an uncommon clinical presentation of advanced malignancy and may occasionally be the first manifestation of an otherwise undiagnosed cancer. It is generally associated with advanced disease and a poor prognosis. Given its rarity, the available evidence is largely limited to case reports and small case series, with considerable heterogeneity in terms of underlying malignancy, clinical presentation, diagnostic work-up, and treatment. We therefore conducted a focused review of the literature to characterize the clinical features, associated malignancies, diagnostic approaches, treatment strategies, and outcomes of patients presenting with malignant priapism. Searches were conducted in PubMed and Scopus using combinations of the terms “malignant priapism”, “penile metastasis”, “penile metastases”, “priapism” and “penile involvement”, with additional screening of the reference lists of relevant articles and reviews to identify further eligible reports. The final literature search was performed on 10 August 2026. Only English-language publications were considered. Duplicate records were identified and removed manually before the final selection of eligible cases. Only cases presenting with priapism in association with malignant penile involvement were considered eligible, whereas reports describing penile metastases without priapism were excluded. A total of 32 individual cases were identified and included in the analysis. The main clinical and oncological characteristics of these patients, including age, primary malignancy, previous knowledge of the underlying cancer, priapism as the first manifestation of malignancy, type of priapism, pattern of penile involvement, treatment and outcome, are summarized in Table 2. Given the rarity of malignant priapism and the heterogeneity of the available literature, the analysis was descriptive and focused on the identification of recurrent clinical patterns rather than on formal statistical comparisons. The findings of this descriptive analysis are presented and discussed in the Discussion section in the context of the present case.

## 4. Discussion

Malignant priapism is a rare urological emergency and typically represents a late manifestation of advanced malignancy. Typical clinical presentations of penile metastases include penile pain, priapism, palpable nodules, ulceration, and urinary symptoms such as hematuria or dysuria [37]. However, in rare cases, malignant priapism may represent the sole initial manifestation, as observed in the present case. It is most commonly associated with pelvic tumors, while penile involvement from extrapelvic primaries, particularly lung cancer, is exceedingly uncommon [4].

Penile metastases from lung cancer have been reported only in a limited number of cases, usually in the context of disseminated disease. In a comprehensive review by Guo et al. [38], fewer than 40 cases of penile metastasis from primary lung cancer were identified, with most patients presenting with widespread metastatic disease at diagnosis. Similar observations have been reported in other case series, confirming the rarity and poor prognosis of this condition [39].

The present case is unusual because priapism was the only initial symptom of an otherwise clinically silent and widely metastatic disease. No pulmonary or systemic symptoms were present at diagnosis, and penile involvement occurred in the absence of visible cutaneous lesions, despite extensive infiltration of the corpora cavernosa. Only isolated cases have been described, in which malignant priapism preceded the diagnosis of the primary tumor [1]. In the present case, the initial MRI showed diffuse corporal abnormalities without a clearly defined focal mass, whereas repeat imaging approximately two months later demonstrated marked progression with more extensive infiltrative features. In retrospect, the initial findings may have represented an early stage of an infiltrative process that was not yet radiologically overt. Alternatively, rapid progression may have occurred during the interval, particularly in the context of the subsequently documented widespread metastatic disease. This evolution highlights that a non-specific initial MRI should not exclude malignant corporal infiltration when symptoms persist or progress despite conventional treatment.

From a differential diagnostic perspective, several possibilities should be considered, including: (1) metastatic carcinoma from a pulmonary primary, (2) metastatic disease from an occult primary tumor of unknown origin, and (3) primary penile malignancy.

Adenosquamous carcinoma of the lung is known as an aggressive histological subtype characterized by rapid growth, early metastasis and inferior survival compared to other non-small cell lung cancers [7]. From a pathological perspective, this case is limited by the absence of a comprehensive immunohistochemical panel and the lack of histological confirmation of the suspected primary site. The immunophenotype was non-specific, and key markers for pulmonary origin, such as TTF-1 and Napsin A, were not available. In addition, the lack of p63 and p40 expression makes the identification of a squamous component less definitive.

Therefore, although morphological features suggested adenosquamous differentiation, the possibility of a poorly differentiated adenocarcinoma cannot be entirely excluded. Based on the overall clinical presentation, imaging findings, and pattern of dissemination, a pulmonary origin is considered the most likely. However, in the absence of histological confirmation, the diagnosis should be regarded as presumptive. Therefore, this case may also be interpreted as metastatic carcinoma of unknown primary with features strongly suggestive of lung origin.

The 32 patients identified in our focused literature review were predominantly older adults, with a mean age of 64.7 years among the 22 patients for whom age was reported (range, 25–86 years). The relatively low age of the two patients with testicular malignancies contributed to lowering the overall mean age, whereas most patients with other primary malignancies were in their sixth to eighth decades of life.

Urological malignancies accounted for the clear majority of primary tumours. Bladder cancer was the most frequently reported primary malignancy, accounting for 12 of 32 cases (37.5%), followed by prostate cancer in 6 cases (18.8%) and renal cell carcinoma in 3 cases (9.4%). Other urological primaries included testicular malignancies (2 cases, 6.3%), urethral squamous cell carcinoma (1 case, 3.1%), and transitional cell carcinoma of the prostate (1 case, 3.1%). Non-urological primary tumours were less frequently represented and included colorectal malignancies in 3 cases overall, comprising two rectal carcinomas and one mucinous adenocarcinoma of the caecum (9.4%), as well as malignant melanoma, malignant glomus tumour/glomangiosarcoma of the corpus cavernosum, and lung adenocarcinoma (one case each, 3.1%). One additional case was described as a high-grade urothelial malignancy without a more precisely specified primary site. Overall, these findings confirm the predominance of genitourinary malignancies in malignant priapism, with bladder and prostate cancer representing the most frequently reported primary tumours. Among non-urological malignancies, colorectal cancer was the most frequently represented group.

In the 22 cases in which the temporal relationship between malignancy and priapism was reported, priapism represented the first manifestation of malignancy in 5 patients (22.7%). When all 32 cases were considered, this corresponded to 15.6% of the overall cohort, while the remaining cases either occurred in patients with a previously known malignancy or had insufficient information to establish the timing. Notably, in several patients, priapism represented the first clinical manifestation of recurrence or systemic progression rather than the initial presentation of the primary tumour.

The diagnostic work-up was heterogeneous and generally included a combination of clinical assessment and imaging, with penile Doppler ultrasonography, magnetic resonance imaging, computed tomography, and, in selected cases, positron emission tomography/computed tomography used to characterize penile involvement and assess the extent of metastatic disease [29]. Histological confirmation was obtained in several cases through biopsy or examination of surgically resected tissue. This variability reflects the absence of a standardized diagnostic pathway for malignant priapism and the fact that diagnostic investigations were often tailored to the clinical context and the suspected primary malignancy.

Treatment was similarly heterogeneous and was largely guided by symptom severity, local disease extent, and the patient’s overall oncological status. Surgical treatment was reported in several cases, with penectomy performed in 11 of the 32 patients (34.4%). These procedures included total, radical, and partial penectomy and were generally undertaken in the setting of advanced or symptomatic penile disease, most commonly with a palliative objective. Other treatment strategies included radiotherapy, systemic chemotherapy or immunotherapy, embolization, and local surgical procedures. In selected patients with isolated penile involvement, local treatment was pursued with the aim of achieving symptom control and local disease control.

Overall, the outcomes were generally poor, consistent with the association between malignant priapism and advanced metastatic disease [4]. Several patients experienced rapid clinical deterioration and died shortly after diagnosis, highlighting the severe prognostic implications of this presentation. The combination of extensive local disease, disseminated malignancy, and limited therapeutic options often resulted in a predominantly palliative approach to management.

From a therapeutic perspective, consistent with previous reports, although new radiation techniques have shown favorable outcomes, penectomy should be considered the last resort in clinical management [30]. In the present case, repeated decompressive procedures failed to provide sustained relief, while progressive necrosis and severe pain ultimately led to total penectomy. The procedure resulted in immediate and complete resolution of genital pain. In our review, penectomy was performed in 11 of 32 reported cases (34.4%), generally in the setting of advanced or symptomatic local disease. These observations suggest that penectomy may provide symptom palliation in selected patients with extensive refractory local disease, although the available literature does not allow conclusions regarding its comparative efficacy.

This case report underscores the importance of considering malignant etiologies in patients presenting with refractory priapism, particularly when corporal induration, progressive or atypical pain, or discordant imaging findings are present. Early imaging and tissue sampling are essential; however, diagnostic certainty may remain limited in the absence of complete histological evaluation.

Limitations of this report include the lack of histological confirmation of the suspected primary lung lesion, the absence of autopsy, and incomplete immunohistochemical characterization. These factors prevent definitive identification of the primary tumor site and necessitate a cautious interpretation of the findings.

## 5. Conclusions

Malignant priapism may represent the first manifestation of advanced metastatic disease. Persistent or recurrent priapism associated with corporal induration, progressive or atypical pain, failure of conventional treatment, or discordant radiological findings should raise suspicion of malignant infiltration and may warrant repeat imaging and tissue sampling. In the present case, carcinoma involving the penile structures was histologically confirmed, whereas pulmonary origin remained a clinicoradiological suspicion. Penectomy provided immediate pain relief in the setting of extensive refractory local disease, although management should be individualized according to disease burden, prognosis, available treatment options, and patient preferences.

## Figures and Tables

**Table 1:** Chronological timeline of clinical events.

**July 2025**	Onset of penile induration followed by development of priapism (initially painless).
**August 2025**	Presentation to the Emergency Department with persistent painful priapism. Initial corporal aspiration and irrigation performed with temporary resolution.
**August 2025**	Penile Doppler ultrasound and laboratory work-up performed; findings consistent with ischemic priapism.
**2 September 2025**	First penile MRI showing no focal mass lesion. Cystoscopy showing no bladder tumours.
**September 2025**	Recurrence of priapism; distal T-shunt performed without sustained detumescence.
**September 2025**	Further recurrence; proximal corporospongiosal shunt performed. Intraoperative biopsy obtained.
**September 2025**	Histology from biopsy suggestive of squamous carcinoma.
**31 October 2025**	Repeat MRI showing progressive infiltrative disease.
**November 2025**	Total-body CT revealing left lung mass and metastatic spread.
**November 2025**	Bronchoscopy with needle aspiration (non-diagnostic).
**13 November 2025**	Total penectomy performed for refractory pain and necrosis.
**Postoperative period**	Symptom relief; complications managed.
**Late November 2025**	Whole-body PET-CT demonstrating widespread metastatic disease.
**A few days later**	Clinical deterioration and death due to respiratory failure.

**Table 2:** Summary of 32 published cases of malignant priapism identified through a focused literature review, detailing patient characteristics, primary malignancy, clinical presentation, mechanism of penile involvement, treatment, and outcome. NR: Not Reported.

	Reference	Age	Primary Tumour	Known Malignancy Before Priapism	First Manifestation of Malignancy	Type	Penile Involvement/Mechanism	Treatment/Outcome
**1**	Berard & Byrne, 1953 [9]	NR	Bladder carcinoma	NR	NR	NR	Penile metastasis	NR
**2**	Okumura et al., 1984 [10]	**45**	Rectal carcinoma	**Yes**	No	NR	**Corpora cavernosa**; metastatic recurrence	NR; pulmonary + local recurrence
**3**	Nezu et al., 1998 [11]	78	Renal cell carcinoma	**No**	**Yes**	NR	**Corpora cavernosa + spongiosum**	Radiotherapy
**4**	Dubocq et al., 1998 [12]	66	Bladder carcinoma	**Yes**	No (first sign of recurrence)	**High-flow**	**Isolated corpora cavernosa metastasis**	Radiotherapy
**5**	Chan et al., 1998—case 1 [13]	NR	Bladder carcinoma	NR	NR	NR	Penile metastasis	NR
**6**	Chan et al., 1998—case 2 [13]	NR	Prostate carcinoma	NR	NR	NR	Penile metastasis	NR
**7**	Hettiarachchi et al., 2001 [14]	**40**	Urethral SCC	NR	NR	NR	**Direct extension** from bulbous urethra to the penile urethra, spongiosum and cavernosa	Systemic chemotherapy; poor prognosis
**8**	Casoli et al., 2002 [15]	NR	Bladder TCC	NR	NR	NR	Penile metastasis	Chemotherapy/cystectomy mentioned; fatal outcome
**9**	Trívez Boned et al., 2004 [16]	NR	Bladder TCC	NR	NR	NR	Penile metastasis	NR
**10**	Inamoto et al., 2005 [17]	86	Testicular cancer	Yes	No (first sign of recurrence)	NR	Penile metastasis	Palliative Penectomy
**11**	Celma Doménech et al., 2008 [18]	NR	Bladder carcinoma	Yes	No	NR	Direct invasion of corpora cavernosa	NR
**12**	Jiang et al., 2009 [19]	25	Testicular tumour	No	No	NR	Penile metastasis	NR
**13**	Okinami et al., 2009 [20]	NR	Malignant melanoma	**Yes**	No	NR	Penile metastasis	**Partial penectomy**; primary disease end-stage
**14**	Park et al., 2009 [21]	**43**	Rectal adenocarcinoma	**Yes**	No	NR	Penile metastases involving corpora cavernosa	**Palliative radiotherapy**; pain relief
**15**	Sönmez et al., 2009 [22]	74	Bladder urothelial carcinoma	**Yes**	**Yes (first systemic manifestation)**	NR	Penile metastasis	**Radiotherapy followed by chemotherapy, poor prognosis**
**16**	Eguíluz Lumbreras et al., 2009 [23]	NR	Bladder TCC	**Yes**	No	NR	Penile metastasis; corpora cavernosa biopsy positive	NR; advanced disease
**17**	Masson-Lecomte et al., 2011 [24]	32	Malignant glomus tumour/glomangiosarcoma	No	**Yes**	**High-flow**	**Primary corpus cavernosum tumour** rather than metastasis	Embolization + Chemotherapy followed by transperineal tumorectomy
**18**	Lin et al., 2011 [25]	**84**	Prostate adenocarcinoma	**Yes**	No	Ischaemic/low-flow	Penile metastasis; cavernosal biopsy	Surgical exploration/biopsy; extensive subsequent metastases
**19**	Ellanti et al., 2011 [26]	60	High-grade urothelial malignancy	No	No	Low-flow	Penile metastasis; **no disseminated disease**	Palliative total penectomy
**20**	Ahmed et al., 2012 [27]	**73**	Bladder urothelial carcinoma	**Yes**	**No (first sign of recurrence)**	NR	Penile metastasis	Palliative penectomy; rapid progression
**21**	Mochizuki et al., 2012 [28]	**70**	Papillary RCC	**No**	**Yes**	**Non-ischaemic/high-flow**	Penile metastasis	**Total penectomy + radical nephrectomy + temsirolimus**; partial remission/stable disease
**22**	Sasikumar et al., 2013 [29]	56	Mucinous adenocarcinoma of caecum	**Yes**	No	NR	Penile metastasis	FDG-PET/CT + trucut biopsy; treatment NR
**23**	**Tabei case 1**, 2023 [30]	**59**	Bladder urothelial carcinoma	**Yes**	No	**Ischaemic/low-flow**	Distal right penile corpora metastasis	**Palliative penectomy**; pain relief; died ~2 months later
**24**	**Tabei case 2**, 2023 [30]	**71**	Bladder urothelial carcinoma	**Yes**	No	**Ischaemic/low-flow**	Glans + corpus spongiosum; metastatic UC	**Penectomy**; hospice; died ~2 months later
**25**	**Tabei case 3**, 2023 [30]	**77**	Prostate adenocarcinoma	**Yes**	No	**Ischaemic/low-flow**	Bilateral corpora cavernosa; local tumour extension	**Radical penectomy** + cystoprostatectomy/orchiectomy; died shortly afterwards
**26**	Tayeh et al., 2020 [31]	**77**	Bladder urothelial carcinoma	**Yes**	No	**Stuttering priapism**	Corpora cavernosa + spongiosum + urethra; isolated penile recurrence	**Total penectomy**; isolated penile metastasis
**27**	Arai et al., 2025 [32]	**75**	Clear-cell RCC	**Yes**	No	**Non-ischaemic**	**Corpus cavernosum metastasis**	Penile artery embolisation + **Radiotherapy**; died ~3 months later
**28**	Zhang et al., 2025 [33]	**76**	Prostate adenocarcinoma	**Yes**	No	**Ischaemic/low-flow**	Penile metastasis	**Palliative penectomy**; pain improved; alive at 5-month follow-up
**29**	Ravichandran et al., 2025 [34]	**70**	Prostate adenocarcinoma	**Yes**	No	NR	Multiple penile metastatic deposits	**Total penectomy + perineal urethrostomy**; subsequently died
**30**	Abou Zahr et al., 2024 [1]	**86**	Lung adenocarcinoma	**No**	**Yes**	**Non-ischaemic/high-flow**	Corpora cavernosa; widespread visceral/bony metastases	No local treatment; systemic chemo/immunotherapy; surveillance of penis
**31**	Savion et al., 1987 [35]	**NR**	Prostate adenocarcinoma	**NR**	**NR**	**NR**	NR	NR
**32**	Rodrigues Netto et al., 1990 [36]	**NR**	Transitional Cell Carcinoma of the Prostate	**NR**	**NR**	**NR**	NR	NR

## Data Availability

The data presented in this study are available upon reasonable request from the corresponding author, due to privacy and confidentiality considerations regarding the patient involved in this case report.

## Abbreviations

The following abbreviations are used in this manuscript:

CK	Cytokeratin
CK7	Cytokeratin 7
CT	Computed Tomography
IHC	Immunohistochemistry
MRI	Magnetic Resonance Imaging
Nx	Regional lymph nodes (not assessed)
p16	Protein p16
p40	Protein p40
p63	Protein p63
pT	Pathological Tumor stage
PET/CT	Positron Emission Tomography/Computed Tomography
PSA	Prostate-Specific Antigen
T3	Tumor stage indicating local extension beyond the organ of origin
